# PEComa of the uterus with coexistence of situs inversus totalis, a case report and literature review

**DOI:** 10.1186/s13000-015-0351-8

**Published:** 2015-08-14

**Authors:** Yang Han, Ting-ting Liu, Xue-shan Qiu, Qing-chang Li, Yi Zhao, Xiao-Yan Pang, En-hua Wang

**Affiliations:** Department of Pathology, The First Affiliated Hospital of China Medical University, Shenyang, China; Department of General Surgery, The First Affiliated Hospital of China Medical University, Shenyang, China; Department of Obstetrics and Gynecology, The First Affiliated Hospital of China Medical University, Shenyang, China

**Keywords:** PEComa, Uterus, Situs inversus totalis

## Abstract

PEComas are a group of very rare mesenchymal neoplasms, which express myogenic and melanocytic markers, such as HMB-45 and actin. Situs inversus totalis represents a complete left to right side transposition of the asymmetrical thoracic and abdominal organs and incorporates dextrocardia. The presence of uterus PEComa in the setting of situs inversus totalis is extremely rare. Here, we report a case of PEComa of uterus with coexistence of situs inversus totalis and review the literatures. To the best of our knowledge this is the fist report of a uterus PEComa patient with situs inversus totalis.

## Background

PEComas are a group of very rare mesenchymal neoplasms, which expresses myogenic and melanocytic markers, such as HMB-45 and actin. The PEComa family of tumors has subsequently grown to include angiomylipoma (AML), clear cell "sugar" tumor of the lung (CCST), lymphangioleiomyomatosis (LAM), and a number of unusual visceral, intraabdominal, soft tissue and bone tumors. Situs inversus totalis (SIT) represents a complete left to right side transposition of the asymmetrical thoracic and abdominal organs and incorporates dextrocardia. It is estimated to occur in between 1:5,000-20,000 adults.

## Case presentation

### Clinical history

A 44-year-old woman visited our hospital reporting menorrhagia and dysmenorrhea with history of nausea and vomiting for weeks.

Ultrasound scan showed the size of the uterus was 8.0 cm × 9.9 cm × 9.8 cm, the thickness of endometrium was 0.6 cm. There was inhomogeneous high echo with star-like color flow can be seen in the posterior wall of the uterus, and the range was 7.2 cm × 5.5 cm. The left ovary was 2.1 cm × 1.3 cm and the right was 2.6 cm × 1.8 cm, with the bilateral boundaries of the ovaries were clear. The bilateral fallopian tubes showed no obvious abnormalities.

Ultrasound scan of abdomen showed the liver located on the left side of abdominal cavity, and the spleen located on the right side. The size and shape of the bilateral kidneys were normal.

CT diagnosis report: The thoracic cage was symmetric. The bilateral lung fields were clear. The size of the heart was normal, dextrocardia. The abdominal organs were mirror reverse, situs inversus viscerum.

**Fig. 1:**
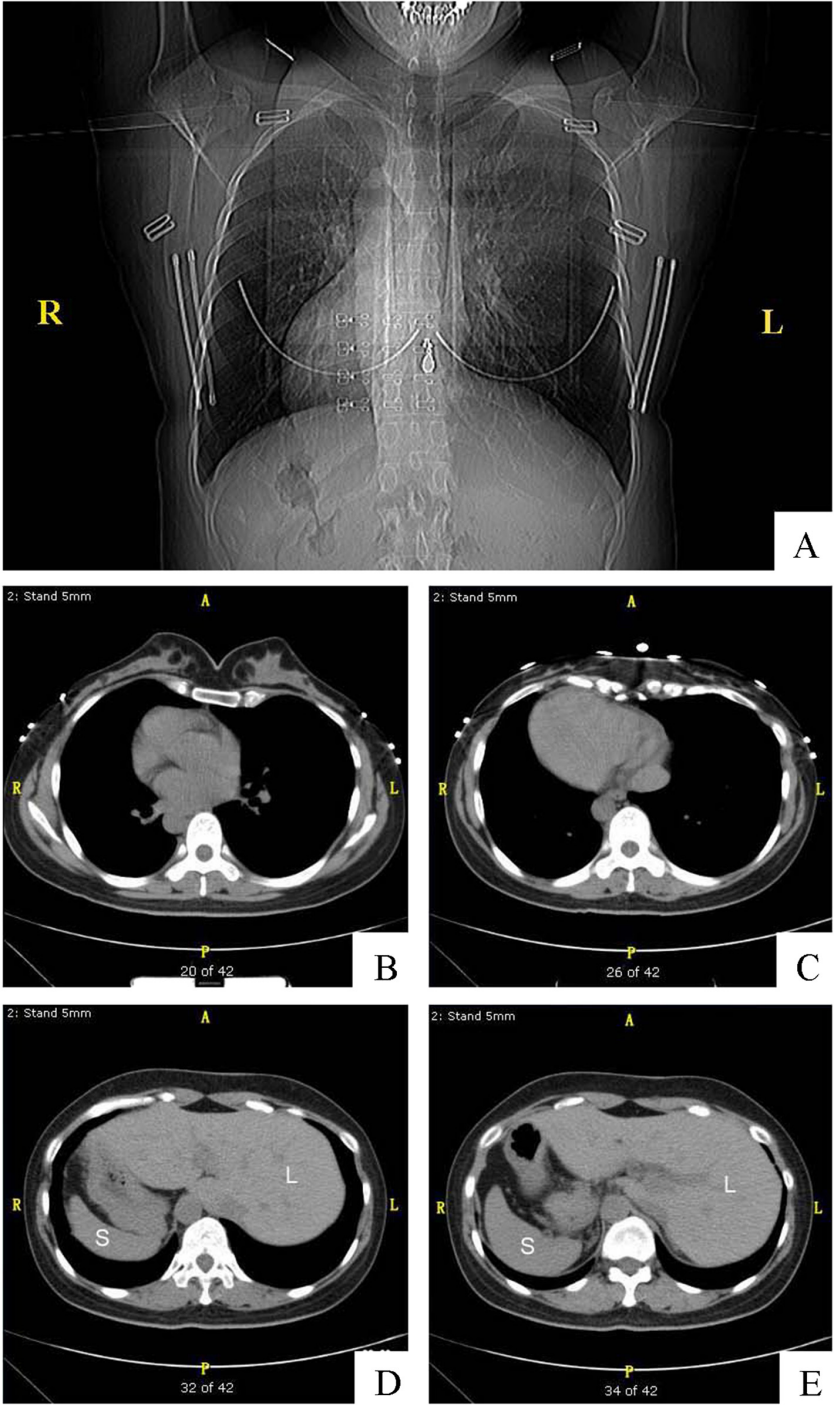
The computed tomography image of the chest demonstrated a normal-sized heart with dextrocardia, right-sided gastric air bubble, aortic knob and descending aorta. The left hemidiaphragm was higher than the right one. This picture was compatible with situs inversus totalis. **a** Thoracic computed tomography scan shows the dextrocardia (**b** and **c**). The computed tomography image of the abdomen. CT scan shows the liver on the left side and spleen on the right, which confirms the presence of situs inversus totalis. Note the situs inversus anatomy of the abdominal organs. L: Liver S: Spleen (**d** and **e**)

Chest and abdominal computed tomography (CT) showed situs inversus totalis (Fig. 1). A total hysterectomy was performed under general anesthesia.

### Pathology

#### Gross

**Fig. 2:**
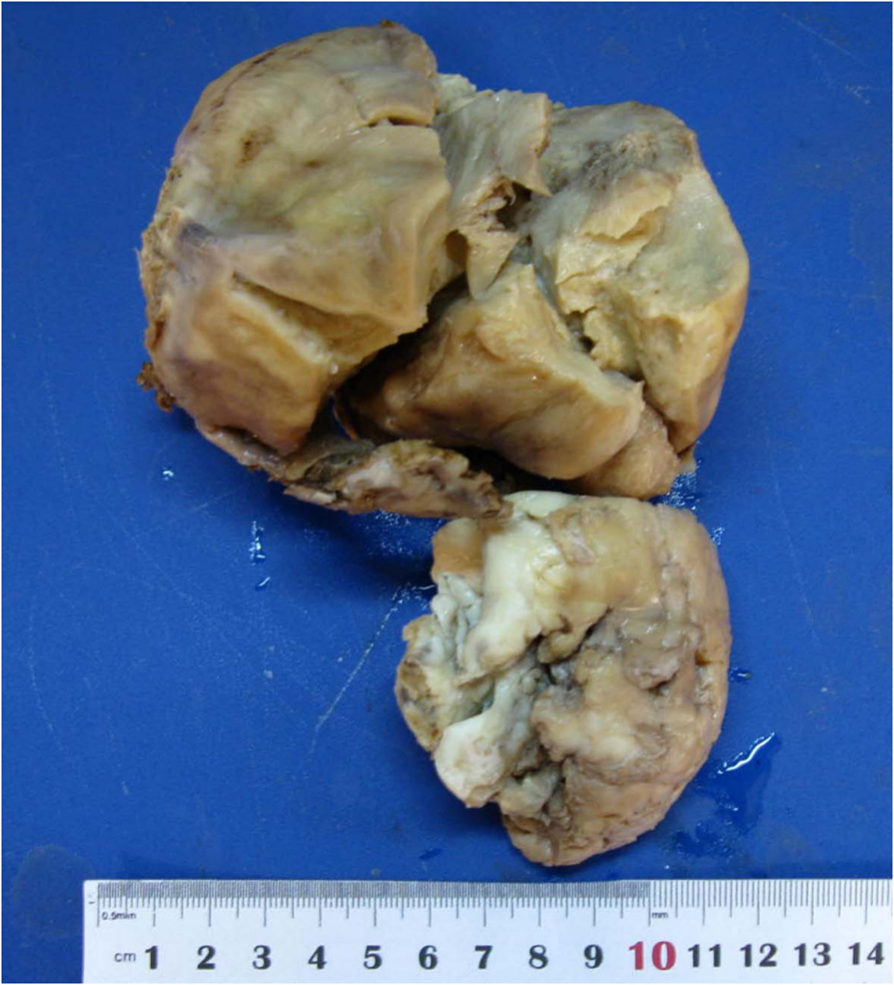
The uterus was 10 cm × 10 cm × 8.5 cm and there was an irregular nodular in the posterior wall of the uterus. The resected specimen of the nodular showed a white and gray, 7.0 cm in maximum diameter, an ill-defined border, and no capsule formation

Macroscopically, the size of the uterus was 10 cm × 10 cm × 8.5 cm and there was an irregular nodular in the posterior wall of the uterus. The resected specimen of the nodular showed a white and gray, 7.0 cm in maximum diameter, an ill-defined border, and no capsule formation (Fig. 2).

#### Histology and immunohistochemistry

The tumor was fixed in 10 % formalin and embedded in paraffin. Several 4-μm sections were cut from each paraffin block. Hematoxylin-eosin (HE) and immunohistochemical (IHC) stains were performed. IHC staining was performed using the streptavidin-peroxidase system (Ultrasensitive; Maxim Inc., Fuzhou, China) according to the manufacturer's instruction. Commercially available prediluted monoclonal antibodies against the following antigens were employed: Vimentin ((V9), 1:200, Maxim), CK ((AE1/AE3), 1:200, Maxim), Melan-A((A103), 1:200, Dako), HMB-45(1:200, Dako), Desmin((D33), 1:200, Dako), SMA((1A4), 1:200, Maxim), ER((SP1), 1:200, Maxim) PR((SP2), 1:200, Maxim), p53((DO-7), 1:200, Maxim), Syn((SP11), 1:200, Maxim), chromogranin-A((DAK-A3), 1:200, Dako), S100((4C4.9), 1:200, Maxim), CD38((F7101), 1:200, Dako), CD138((M115), 1:200, Dako), and Ki-67 ((MIB-1), 1:200, Maxim). The immune reactions were visualized with the use of DAB as the chromogen (Sigma-Aldrich Co, St Louis, Mo, USA). All internal and external controls worked appropriately.

**Fig. 3:**
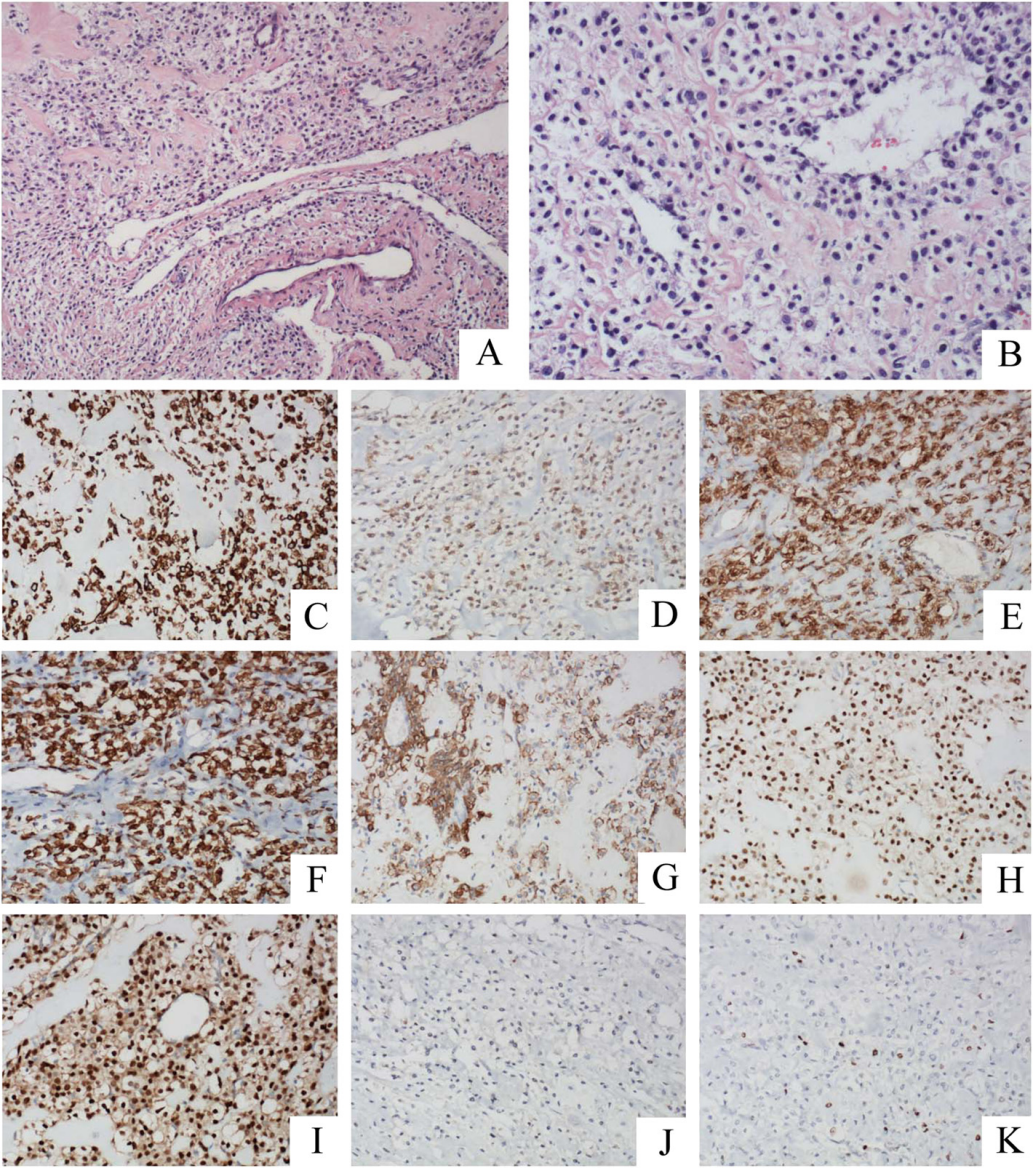
The tumor consisted of round and polygonal cells with clear to eosinophilic granular cytoplasm. The tumor cell nuclei showed hyperchromasia, nuclear enlargement (**a** and **b**). The tumor cells were positive for Vimentin (**c**), Melan-A (**d**), HMB-45 (**e**), Desmin (**f**), SMA (**g**), ER (**h**), PR (**i**), but negative for AE1/AE3 (**j**). Ki-67 showed a nuclear positivity in approximately 10 % of cells (**k**)

By histology, the tumor consisted of round and polygonal cells with clear to eosinophilic granular cytoplasm. The tumor cells proliferated in a honeycomb-like appearance and often were arranged in a radial fashion around blood vessels. The tumor cell nuclei showed hyperchromasia, nuclear enlargement, and small nucleoli (Fig. 3a and b). The tumor cell showed slight to moderate nuclear atypia, with no obvious mitosis or tumor necrosis. There was no lymphatic or vascular invasion.

By immunohistochemistry, the tumor cells were positive for Vimentin (Fig. 3c), Melan-A (Fig. 3d), melanoma-associated antigen (HMB-45) (Fig. 3e), Desmin (Fig. 3f), smooth muscle actin (SMA) (Fig. 3g), estrogen receptor (ER) (Fig. 3h), progesterone receptor (PR) (Fig. 3i) and p53, but negative for AE1/AE3 (Fig. 3j), Synaptophysin, Chromogranin A, S-100, CD38, and CD138. The proliferation marker Ki-67 showed a nuclear positivity in approximately 10 % of cells (Fig. 3k).

### Results

A uterus PEComa was diagnosed on the basis of the above findings. The patient received only surgical resection. She is undergoing regular physical examination and remains free of disease at 2 months after operation.

## Discussion

The concept of the perivascular epithelioid cell (PEC) was proposed by Bonetti etal. in 1992 [1]. The cells constantly present in a group of tumors called PEComas but do not exist in the normal organs and tissues. PEC expresses myogenic and melanocytic markers, such as actin and HMB45. The PEComa was proposed by Zamboni etal. in 1996 to describe a very rare mesenchymal neoplasms, which was accepted by the World Health Organization (WHO) in 2002 [2]. The PEComa family of tumors include angiomylipoma (AML), clear cell "sugar" tumor of the lung (CCST), lymphangioleiomyomatosis (LAM), and a number of unusual visceral, intraabdominal, soft tissue and bone tumors, which have been described under a variety of names, including clear cell myomelanocytic tumor of the falciform ligament/ligamentum teres (CCMMT), abdominopelvic sarcoma of perivascular epithelioid cells, and primary extrapulmonary sugar tumor, among others [3]. PEComas have been reported in various organs, such as kidney, liver, lung, pancreas, thigh, breast, gastrointestinal tract, prostate, uterus, and uterine cervix, cardiac septum, falciform ligament, ect. The tumors usually show immunoreactivity for both melanocytic (HMB-45 and/or Melan-A) and smooth muscle (actin and/or desmin) markers. Several reports described TFE3 expression in PEComas [4, 5]. Folpe et al. concluded the diagnostic criteria of PEComas. Benign criteria was, no worrisome features (the diameter of the tumor < 5 cm, non-infiltrative, non-high nuclear grade and cellularity, mitotic rate ≤ 1/50HPF, no necrosis, no vascular invasion); uncertain malignant potential criteria was, nuclear pleomorphism/multinucleated giant cells only or size > 5 cm only; and malignant criteria was, two or more worrisome features (>5 cm, infiltrative, high nuclear grade and cellularity, mitotic rate > 1/50HPF, necrosis, vascular invasion) [6].

The differential diagnosis of soft tissue and gynecologic PEComas is fairly broad and predominantly guided by the morphology (epithelioid vs. spindled) and location of the tumor. Because of their expression of HMB-45 and/or melan-A, PEComas are frequently confused with both conventional melanoma and clear cell sarcoma. Some of the morphologic features of PEComas, including the admixture of spindled and epithelioid forms, the occasionally prominent nucleoli, and the presence of multinucleated cells, are also seen in melanoma and clear cell sarcoma. In most cases, melanoma/clear cell sarcoma can be distinguished from PEComa by their strong expression of S-100 protein and smooth muscle actin nonimmunoreactivity [6].

Complete resection is the preferred treatment for PEComa at present, but for the specific scope of operation is no definite conclusion. Most authors believe that hysterectomy and adnexectomy are the most direct and effective means of treatment. But for the malignant PEComa of uterus, pelvic lymph nodes dissection and postoperative adjuvant chemotherapy remains controversial. The plan of chemotherapy is still in study and discussion.

Situs inversus totalis (SIT) is a condition with left-to-right reversal of the viscera combined with dextrocardia and individual organs present a symmetrical mirror image morphology. It is frequently complicated by concurrent abnormalities in the cardiovascular system and the hepatobiliary system. The prevalence of SIT is estimated to occur in between 1:8000 and 1:25000 [7, 8]. No racial predilection exists and the male-to-female incidence is 1:1. Computed tomography and magnetic resonance imaging are the preferred examinations for the definitive diagnosis of SIT. The recognition of SIT may help us avoid mishaps at surgery or other interventions that result from the failure to recognize reversed anatomy.

## Conclusions

In summary, we reported a unique case of PEComa of uterus with coexistence of situs inversus totalis and review the literatures. The presence of uterus PEComa in the setting of SIT is extremely rare. To the best of our knowledge this is the first report of a uterus PEComa patient with SIT. There is no evidence that the presence of SIT predisposes one to develop PEComa. The clinicians should keep in mind that uterus PEComa may accompany SIT.

## Consent

Written informed consent was obtained from the patient for publication of this Case report and any accompanying images. A copy of the written consent is available for review by the Editor-in-Chief of this journal.
